# The Latin American Association of Cardiac and Endovascular Surgery statement regarding the recently released 2020 ACC/AHA Guidelines for the Management of Patients with Valvular Heart Disease

**DOI:** 10.21470/1678-9741-2021-0952

**Published:** 2021

**Authors:** Victor Dayan, Ovidio A. Garcia-Villarreal, Alejandro Escobar, Javier Ferrari, Eduard Quintana, Mateo Marin-Cuartas, Rui M S Almeida

**Affiliations:** 1 Centro Cardiovascular Universitario, Montevideo, Uruguay.; 2 Mexican College of Cardiovascular and Thoracic Surgery, Mexico City, Mexico; 3 Universidad CES, Medellin, Colombia.; 4 Colegio Argentino de Cirujanos Cardiovasculares, Buenos Aires, Argentina.; 5 Cardiovascular Surgery Department, Hospital Clinic Barcelona, Barcelona, Spain.; 6 University Department of Cardiac Surgery, Leipzig Heart Center, Leipzig, Germany.; 7 Department of Cardiothoracic Surgery, Stanford University, Stanford, USA.; 8 University Center Assis Gurgacz, Cascavel, PR, Brazil.

Scientific evidence in the cardiological arena has progressed enormously in the last couple of years. It is a huge challenge for respected societies such as AHA and ACC to undertake the burden of providing to the world their recommendations for clinical practice based on this evidence. The Latinamerican Association of Cardiac and Endovascular Surgery (LACES) would like to thank the authors involved in such a task.

As a growing association that represents an economic and healthcare reality that is different from others, we have decided to carefully select guidelines that consider our socio-economic situation. As such, in this statement, we will highlight the aspects of the recently released AHA/ACC Guidelines for the Management of Patients with Valvular Heart Disease 2020 with which we disagree intending to support Latin-American surgeons in their practice^[1]^.

## AORTIC STENOSIS

Trials on TAVI and SAVR have been constructed based on surgical risk; for this reason, the previous AHA/ACC Guidelines for the Management of Patients with Valvular Heart Disease based its recommendation for the type of intervention on surgical risk.

Although we support the concept that age, expected survival and valve durability are the cornerstone for Patient-Heart Team discussion, trials have not evaluated outcomes based on age. Furthermore, the age range used to support TAVI is well below the mean age of the low-risk trials (73 years old for PARTNER 3 and 74 years old for EVOLUT Low Risk) and there is absolutely no reference to support this range defined by the authors.

Therefore, LACES considers an important methodological flaw subject to high risk of reversal, to recommend as Class of Recommendation (COR) I Level of Evidence A (the highest imprimatur of guideline recommendations) any indication for TAVI or SAVR based on age. We consider this discrepancy of utmost importance since AHA/ACC recommendations will guide treatment and provide legal framework in several countries of thousands of patients which in this case is devoid of scientific evidence.

LACES does not support any COR I level of evidence A recommendation, which is NOT supported by large randomized control trials. Large randomized control trials have been constructed based on surgical risk. Therefore, we do not support any recommendation on TAVI or SAVR based on age.

The authors have clearly stated the importance of life expectancy and valve durability to help decide the best strategy. Nonetheless, there is no mention about the long-term risk of paravalvular leak or permanent pacemaker implantation on long-term survival in low-risk and young patients. PARTNER 2 trial has shown patients with a mild paravalvular leak to have worse survival at 5 years (*P*=0.06) than patients with none or trace^[2]^. We believe this issue to be as important as valve durability and therefore be seriously incorporated in the decision for patients with longer than 5 years life expectancy. Until there are no data on its detrimental effect, we do not believe that it is safe to recommend TAVI in patients with >5 years of life expectancy. Since there is no evidence longer than a median of 5 years of follow-up, to support the safety of TAVI in intermediate- and low-risk patients and also regarding the detrimental effect of paravalvular leak, LACES does not support any COR I for TAVI in patients with a life expectancy longer than 5 years.

Surgical risk defined by the current guidelines is another novel topic in which this association has a different view. High risk has been defined by the current guidelines as any of the following:

- STS >8%,
- > or =2 indices in frailty,
- 1 to 2 organ system compromise not to be improved postoperatively and
- possible procedure-specific impediment.

Organ system compromise has been defined as cardiac dysfunction (severe LV systolic or diastolic dysfunction or RV dysfunction, fixed pulmonary hypertension); kidney dysfunction (chronic kidney disease, stage 3 or worse); pulmonary dysfunction (FEV1 < 50% or DLCO2 < 50% of predicted); central nervous system dysfunction (dementia, Alzheimer’s disease, Parkinson’s disease, cerebrovascular accident with persistent physical limitation); gastrointestinal dysfunction (Crohn’s disease, ulcerative colitis, nutritional impairment or serum albumin <3.0); cancer (active malignancy); and liver dysfunction (any history of cirrhosis, variceal bleeding or elevated INR in the absence of VKA therapy).

There is no reference to support defining high surgical risk under these conditions and we consider the organ system compromisse definition to be very broad resulting probably in a high percentage of patients in this category who will receive a treatment for which there is no evidence to support superiority.

LACES considers that surgical risk stratification should continue to be based on validated scores that result from complex statistical methods and therefore do not support defining high surgical risk based on criteria that do not derive from big data adjusted survival analyses.

Current guidelines have excluded completely the option of SAVR in patients at high surgical risk. Even more, the authors have provided similar recommendations for high surgical risk and prohibitive surgical risk. We consider that this recommendation is unacceptable and our association will not support nor endorse it for the following reasons. Trials have specifically evaluated separately each of these surgical risks (PARTNER 1A and 1B), providing strong and solid evidence based on the population of patients included. PARTNER 1A compared SAVR with TAVR in patients at high risk and PARTNER 1B compared medical treatment and TAVR in patients with prohibitive surgical risk.

Therefore, the conclusion and subsequent guideline recommendations should be based on the population and comparative groups involved. We support palliative care in patients with prohibitive surgical risk in whom TAVI is not feasible.

In patients at high surgical risk, no evidence shows that TAVI is superior to SAVR. The actual evidence is that TAVI is not inferior to SAVR in high risk and, therefore, guidelines recommendation giving Level of Evidence A should reflect this.

Current evidence shows TAVR to be non-inferior to SAVR in patients at high risk; therefore, LACES considers both options to have the same level of recommendation. LACES does not support giving the same recommendation in patients at high and prohibitive risk.

## FUNCTIONAL MITRAL REGURGITATION

After careful consideration of the new 2020 AHA/ACC clinical guidelines for valvular heart disease, we have found several points concerning the recommendations for using transcatheter edge-to-edge mitral valve (MV) repair, as a treatment in the setting of functional mitral regurgitation (FMR), with which we are totally at odds. If CABG is needed, then surgery is indicated as the COR IIA. However, the main disagreement is concerning patients not undergoing CABG.

In these new guidelines, transcatheter edge-to-edge MV repair is considered as a COR IIA, if the case has severe mitral regurgitation (MR) stage D (Rvol >60 ml, RF >50%, EROA >0.4 cm^2^), left ventricular ejection fraction (LVEF) <50%, if symptoms persist on optimal GDMT, with MV anatomy as favourable, LVEF 20-50%, left ventricular end diastolic diameter (LVEDD) >70 mm, pulmonar systolic arterial pressure (PSAP) <70 mmHg. Recommendation has been based mainly on data from the COAPT trial.

First, we need to say is that FMR is not a valvular disease, but a final LV condition leading to heart failure. Every single one of the attempts on the MV (surgical as well as percutaneous) will be just a palliative measure to alleviate the MR. Prognosis remains unaltered, while the quality of life or freedom from symptoms can be improved using any treatment directed to mitigate or even eliminate MR, regardless of the approach. MR repair using restrictive annuloplasty is the more reproducible technique for this purpose. Nevertheless, there has been a great concern because of the MR recurrence in the short term.

While it is true that surgical annuloplasty might not be optimal for cases with FMR, it is also true that there are some other surgical options on the MV, which can be highly recommended, as a definite treatment directed to stop the further dilation and remodelling of the LV. MV replacement is another surgical choice.

However, 1 consideration of paramount importance is that all these foregoing facts apply the same, regardless of whether the approach is surgical or percutaneous.

Moreover, when comparing the results of the COAPT trial (Abbott Funded) with the MITRA-FR trial (French Ministry of Health and Research National Program Funded), both trials differed considerably in the primary outcome^[3,4]^. Besides, a recently published post hoc analysis of a subgroup of patients from the MITRA-FR trial who met COAPT inclusion criteria, transcatheter edge-to-edge MV repair, failed to show superiority over optimal medical therapy. Therefore, longer-term outcomes are required, as well as additional trials.

In the light of the aforementioned details, LACES does not support COR IIA for transcatheter edge-to-edge MV repair, in the presence of F MR. The contradicting outcomes between the Only 2 trials, which have evaluated this technique and the limited 2-3 years of data from the COAPT trial, do not justify the wide expectations of a COR IIA for the percutaneous approach.

Concerning FMR, all the available long-term information is coming from surgical experience, with a significant long-term follow-up of up to 14 years or even longer. From this experience, it has been so very clear that the most powerful predictor for failure after edgeto-edge MV repair is the lack of an annuloplasty ring.

As it stands now in the current version, the transcatheter edgeto-edge MV repair therapy is a ringless technique and therefore longer-term outcomes are needed before we are able to evaluate the safety of a ringless technique.

In regard with comparison to mitral surgery, the EVEREST II trial included 27% of patients with FMR^[5]^. The primary outcome (freedom from death, surgery in the percutaneous group, MR 3+ or 4+, surgery in the repair group) at 1, 2 and 5 years was significantly worse for percutaneous edge-to-edge group. There is no other RCT, which evaluates long-term outcomes of surgery *versus* transcatheter edge-to-edge.

Considering the worse long-term outcomes of the transcatheter edge-to-edge (EVEREST II trial) and absence of long-term safety using a ringless technique, LACES does not support a higher level of COR for the percutaneous approach compared with surgery. We believe that careful evaluation of surgical risk by a Heart Team should define the best approach.

Finally, we believe that one of the main drivers of big societies like AHA, ACC, ESC and EACTS is to thrive to achieve excellence in the treatment of cardiovascular disease worldwide. Scientific societies should acknowledge this to be worldwide leaders in the field.

When guidelines start to diverge from a critical assessment of the scientific evidence with unsupported extrapolations, they lose reliability, practice turns arbitrary and leaders are lost.

This statement has been submitted to several journals to achieve worldwide diffusion of the position of our Association.

## References

[1] Otto CM, Nishimura RA, Bonow RO, Carabello BA, Erwin 3rd JP, Gentile F (2020). 2020 ACC/AHA Guideline for the Management of Patients With Valvular Heart Disease: Executive Summary: A Report of the American College of Cardiology/American Heart Association Joint Committee on Clinical Practice Guidelines. Circulation.

[2] Leon MB, Smith CR, Mack MJ, Makkar RR, Svensson LG, Kodali SK, PARTNER 2 Investigators (2016). Transcatheter or surgical aortic-valve replacement in intermediate-risk patients. N Engl J Med.

[3] Stone GW, Lindenfeld J, Abraham WT, Kar S, Lim DS, Mishell JM, COAPT Investigators (2018). Transcatheter mitral-valve repair in patients with heart failure. N Engl J Med.

[4] Obadia JF, Messika-Zeitoun D, Leurent G, Iung B, Bonnet G, Piriou N, MITRA-FR Investigators (2018). Percutaneous repair or medical treatment for secondary mitral regurgitation. N Engl J Med.

[5] Feldman T, Kar S, Elmariah S, Smart SC, Trento A, Siegel RJ, EVEREST II Investigators (2015). Randomized comparison of percutaneous repair and surgery for mitral regurgitation: 5-year results of EVEREST II. J Am Coll Cardiol.

